# Early Detection of Major Fetal Structural Anomalies in the First Trimester: A Retrospective Single-Center Study in an Unselected Population

**DOI:** 10.3390/jcm15176560

**Published:** 2026-08-25

**Authors:** Maciej Korniluk, Andrzej Korniluk, Grzegorz Szewczyk

**Affiliations:** 1 Department of Obstetrics, Gynecology and Gynecological Oncology, St. John Paul 2nd Mazovia Regional Hospital in Siedlce, 08-110 Siedlce, Poland; 2 Institute of Health Sciences, University of Siedlce, Konarskiego 2, 08-110 Siedlce, Poland; 3 Department of Biophysics, Physiology and Pathophysiology, Medical University of Warsaw, Żwirki i Wigury 61, 02-091 Warsaw, Poland

**Keywords:** first-trimester ultrasound, fetal structural anomalies, early prenatal detection, congenital heart defects, acrania/exencephaly, multiple anomalies, prenatal diagnosis

## Abstract

**Background**: The first-trimester ultrasound (11 + 0 to 13 + 6 weeks) is primarily used for aneuploidy screening, yet its potential for early identification of major fetal structural anomalies in an unselected population continues to be explored. This study evaluated the clinical yield and feasibility of a systematic first-trimester fetal anatomy assessment in a regional setting. **Methods**: This retrospective single-center study included 500 consecutive singleton pregnancies undergoing routine first-trimester ultrasound between 11 + 0 and 13 + 6 weeks of gestation. All examinations were performed by a single certified sonographer using an extended anatomical protocol according to ISUOG and FMF guidelines. The study center serves a regional population of approximately 400,000 inhabitants in eastern Mazovia, Poland. Cases with suspected anomalies underwent a detailed diagnostic work-up, including a targeted anomaly scan, early fetal echocardiography, and invasive genetic testing when clinically indicated. **Results**: Major fetal structural anomalies were suspected in 17 out of 500 fetuses (3.4%) during the first-trimester scan. Of the 17 suspected cases, 14 were confirmed on subsequent evaluation (14/17, 82.4%; 95% CI 56.6–96.2%), two were false-positives (11.8%), and one patient was lost to follow-up. Four fetuses (23.5%) presented with multiple anomalies involving different organ systems, with congenital heart defects being the most prevalent. A notable observational cluster of three cases of acrania/exencephaly was identified. Remarkably, only three of the confirmed affected cases (21.4%) presented with a nuchal translucency measurement above the 95th percentile. **Conclusions**: Integration of a systematic first-trimester fetal anatomy assessment allowed the identification of suspected major structural anomalies in 3.4% of fetuses in an unselected regional population. With a confirmation rate of 82.4% among screen-positive cases, these findings demonstrate the clinical feasibility of detailed first-trimester anatomical assessment in a regional setting.

## 1. Introduction

Congenital structural anomalies represent a major cause of perinatal morbidity and mortality worldwide [1]. Global epidemiological data indicate that major structural defects affect approximately 3–4% of all pregnancies [2]. This prevalence is consistently reflected in prospective cohort studies and systematic reviews evaluating first-trimester ultrasound screening [3,4,5,6], including evaluations in low risk and unselected populations [7]. However, a slightly lower prevalence (approximately 3%) is observed in post-natal surveillance databases among live births due to spontaneous intrauterine losses and early terminations of pregnancy for fetal anomaly (TOPFA) [2,8]. Within Europe, standardized epidemiological reference rates encompassing live births, stillbirths, and TOPFA are consistently provided by the European Surveillance of Congenital Anomalies (EUROCAT) network [9].

In developed nations, the detailed mid-trimester ultrasound scan performed between 18 + 0 and 22 + 6 weeks of gestation remains the established gold standard, identifying roughly 60–70% of major structural anomalies [10]. Nevertheless, by the end of the first trimester, the vast majority of fetal organogenesis is already complete. Driven by rapid advances in ultrasound technology and high-resolution imaging, a comprehensive anatomical survey has become increasingly feasible as early as 11 + 0 to 13 + 6 weeks of gestation [5]. Extensive contemporary literature, encompassing large screening cohorts and multi-center systematic reviews, has demonstrated that a substantial proportion of severe, lethal, or major fetal anomalies can be reliably identified at this early developmental stage [4,6,11,12,13]. This creates an opportunity for timely parental counseling, and more individualized pregnancy management [3,7].

International guidelines issued by the International Society of Ultrasound in Obstetrics and Gynecology (ISUOG) and the Fetal Medicine Foundation (FMF) endorse a basic fetal anatomical survey during the routine 11–14 week scan [14,15]. However, extended first-trimester anatomical protocols have not yet been widely integrated into routine clinical practice, particularly within unselected, low-risk populations. Furthermore, the majority of published evidence regarding the diagnostic performance of early anomaly screening originates either from high-risk cohorts or from tertiary referral centers with specialized maternal-fetal medicine units [16]. Consequently, robust real-world data on the performance of systematic anatomical protocols in unselected populations remain limited [17].

In Poland, as in many other Central and European countries, the second-trimester scan continues to represent the formal, guideline-based cornerstone of prenatal structural anomaly screening [18]. However, with the increasing availability of high-end ultrasound systems in regional hospitals and a growing number of operators holding international certifications, extended first-trimester anatomical assessments are gradually gaining clinical ground. Despite this increasing adoption, high-quality evidence is limited.

The present retrospective study sought to address this gap in the literature by evaluating the clinical yield and feasibility of a systematic first-trimester fetal anatomy assessment using a standardized anatomical protocol in a single regional center. To evaluate this approach, we reviewed 500 first-trimester ultrasound scans performed in an unselected cohort of singleton pregnancies. The primary objective was to determine the prevalence of suspected major structural anomalies and their definitive confirmation rate on subsequent mid-trimester or postnatal evaluations.

## 2. Materials and Methods

### 2.1. Study Design and Population

This retrospective single-center study included 500 consecutive singleton pregnancies undergoing routine first-trimester ultrasound screening from December 2019 to December 2021 at a regional hospital in eastern Mazovia, Poland. The hospital serves an unselected, non-referral regional population.

### 2.2. Inclusion and Exclusion Criteria

Inclusion criteria were defined as a singleton live pregnancy with a gestational age between 11 + 0 and 13 + 6 weeks—confirmed by fetal crown-rump length (CRL) measurement ranging from 45.0 mm to 84.0 mm—where the ultrasound examination was performed according to a standardized protocol by a certified operator. Exclusion criteria included multiple pregnancies and examinations performed outside the recommended gestational window or established protocol.

### 2.3. Ultrasound Examination Protocol

All ultrasound examinations were performed by a single sonographer holding active certifications from both the Fetal Medicine Foundation (FMF; FMF ID: 75770) and the Polish Society of Gynecologists and Obstetricians (PTGiP), possessing over 20 years of experience in obstetric ultrasonography and more than 10 years dedicated specifically to first-trimester fetal anomaly screening.

Examinations were conducted using a high-resolution GE Voluson E8 ultrasound system (GE Healthcare, Chicago, IL, USA). Transabdominal imaging was carried out using a C2-9-D (3–9 MHz) curved array transducer, and transvaginal imaging was performed with an RIC6-12-D (5–12 MHz) volume transducer when maternal habitus or fetal position restricted optimal visualization. Color and power Doppler imaging were systematically utilized in all examinations for a detailed evaluation of cardiac morphology and fetal circulation. The average duration of each comprehensive ultrasound examination ranged from 30 to 45 min.

**Figure 1:**
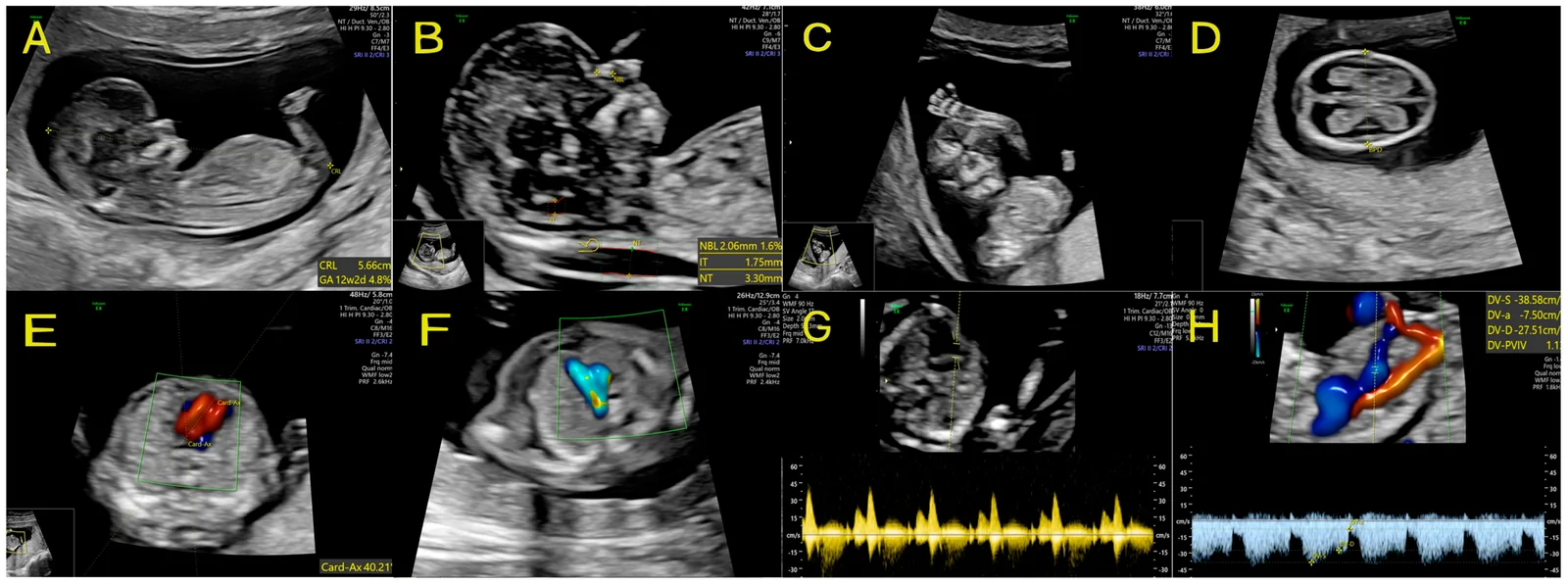
Representative ultrasound images demonstrating the first-trimester screening (11 + 0 to 13 + 6 weeks) in the anatomical protocol used in the study. (**A**) **Crown-rump length (CRL) measurement:** strict midsagittal view of the fetus in a neutral position with horizontal calipers placed from the crown to the rump. (**B**) **Midsagittal view of the fetal face:** assessment of the profile demonstrating correct magnification for nuchal translucency (NT) measurement, presence of the nasal bone (NB), and clear visualization of the intracranial translucency (IT) in the posterior fossa. (**C**) **Retro-nasal triangle view:** coronal scan of the fetal face used to evaluate the primary palate and exclude cleft lip/palate. (**D**) **Transverse view of the brain:** axial plane demonstrating the normal, symmetrical "butterfly sign" formed by the choroid plexuses within the lateral ventricles. (**E**) **Four-chamber view (4CV) of the heart:** apical projection at the level of the fetal thorax demonstrating cardiac axis symmetry and chamber balance. (**F**) **Three-vessel and trachea (3VT) view:** axial upper mediastinal plane showing the normal alignment and spatial relationship of the pulmonary artery, aorta, and superior vena cava relative to the trachea. (**G**) **Axial plane of the fetal chest showing placement of the pulsed Doppler sample volume for tricuspid Doppler flow assessment. Color Doppler assessment of tricuspid valve flow:** interrogation of ventricular inflow to confirm the absence of significant tricuspid regurgitation. (**H**) **Parasagittal view of abdomen and chest in color Doppler assessment of ductus venosus flow:** high-resolution color Doppler visualization evaluating the classic triphasic waveform profile.

The first-trimester protocol was executed in strict accordance with standardized anatomical guidelines. The specific structures and parameters evaluated within this anatomical protocol are summarized in Table 1 and Figure 1.

All captured images, cine loops, and biometric measurements were stored in the Astraia software database version K12112 (Astraia Software GmbH, Munich, Germany) with standardized structured reporting. In cases of a suspected major fetal anomaly (screen-positive cases), patients were referred to a tertiary fetal medicine center for confirmatory expert ultrasound examination and/or early fetal echocardiography. When clinically indicated, patients were offered invasive prenatal diagnostic testing. Definitive outcome confirmation was obtained via second-trimester ultrasound scans and/or pediatric clinical evaluations. Cases lacking definitive mid-trimester or postnatal follow-up were explicitly classified as lost to follow-up and conservatively retained in the denominator for the confirmation rate analysis (14/17, 82.4%).

### 2.4. Combined First-Trimester Screening and Risk Assessment

In all cases, the combined risk for the most common chromosomal abnormalities (trisomies 21, 18, and 13) was calculated using the standardized FMF algorithm based on maternal history, maternal age, ultrasound (NT), and biochemical parameters (free β-hCG, PAPP-A) [19]. Pregnancies were stratified into risk categories according to national guidelines [20]. In cases classified as high risk, patients were offered invasive diagnostic testing via chorionic villus sampling (CVS) or amniocentesis. For pregnancies categorized as intermediate risk, non-invasive prenatal testing (NIPT) utilizing cell-free DNA (cfDNA) was recommended. All clinical management decisions were made following detailed, non-directive counseling regarding the benefits, technical limitations, and specific risks of each diagnostic pathway.

Additionally, the risk of preeclampsia, fetal growth restriction (FGR), and spontaneous preterm birth was calculated concurrently using the FMF multi-parametric algorithm [21]. Women identified as high risk for preeclampsia received appropriate pharmacological prophylaxis, consisting of acetylsalicylic acid at a dose of 150 mg daily, initiated before 16 weeks of gestation [22]. Major structural anomalies were defined according to the European Surveillance of Congenital Anomalies (EUROCAT) criteria as those presenting significant medical, surgical, cosmetic, or functional consequences [9].

### 2.5. Maternal Characteristics and Data Collection

Maternal age, parity, body mass index (BMI), mode of conception (spontaneous versus assisted reproductive technology), and a comprehensive medical and obstetric history—including chronic diseases, prior miscarriages, preterm deliveries, and recurrent fetal anomalies—were obtained directly from the patient during the initial intake interview and supplemented via electronic medical records.

### 2.6. Statistical Analysis

Data management and analysis were performed using Microsoft Excel (Microsoft 365, Microsoft Corporation, Redmond, WA, USA) and Jamovi software (version 2.3, The Jamovi Project, Sydney, Australia). Descriptive statistics were used to summarize the baseline cohort: continuous variables are presented as mean ± standard deviation (SD), and categorical variables are expressed as absolute frequencies and percentages. For selected comparisons between categorical groups, Fisher’s exact test was applied. For screen-positive cases, the confirmation rate (analogous to positive predictive value, PPV) was calculated as the proportion of confirmed major anomalies among all 17 suspected cases. Under a conservative approach, the single case lost to follow-up was retained in the denominator (14/17). The exact Clopper-Pearson method was applied to derive the 95% confidence interval (95% CI) for proportions. Sensitivity analyses under alternative assumptions (excluding the lost-to-follow-up case or classifying it as a false-positive) were also performed. Because systematic long-term follow-up of all screen-negative pregnancies in the entire cohort (*n* = 500) was not available, formal diagnostic performance parameters requiring false-negative rates (such as sensitivity, specificity, and negative predictive value) were not calculated. A two-sided *p*-value *< 0.05* was considered statistically significant.

### 2.7. Ethical Approvals

The study protocol was reviewed and approved by the Local Bioethics Committee of Institute of Health Sciences, University of Siedlce (Decision No. 15/2026). Due to the retrospective nature of the study and complete anonymization of all patient datasets, the requirement for individual informed consent for analysis and publication was formally waived.

## 3. Results

### 3.1. Study Population and Baseline Characteristics

The final analysis included 500 consecutive singleton pregnancies undergoing routine first-trimester ultrasound examination within an unselected screening population. The baseline maternal and clinical characteristics of the study cohort are detailed in Table 2. The mean maternal age was 30.7 ± 5.1 years, and the mean pre-pregnancy body mass index (BMI) was 24.8 ± 4.7 kg/m^2^. The vast majority of pregnancies were conceived spontaneously (99.6%), and multiparous women constituted 68.8% (*n* = 344) of the study population.

### 3.2. Prevalence and Spectrum of Suspected Anomalies

Major fetal structural anomalies were suspected in 17 out of 500 fetuses (3.4%; 95% CI: 2.0–5.4%) during the first-trimester ultrasound scan. The spectrum of suspected anomalies and their outcomes are presented in Table 3.

**Figure 2:**
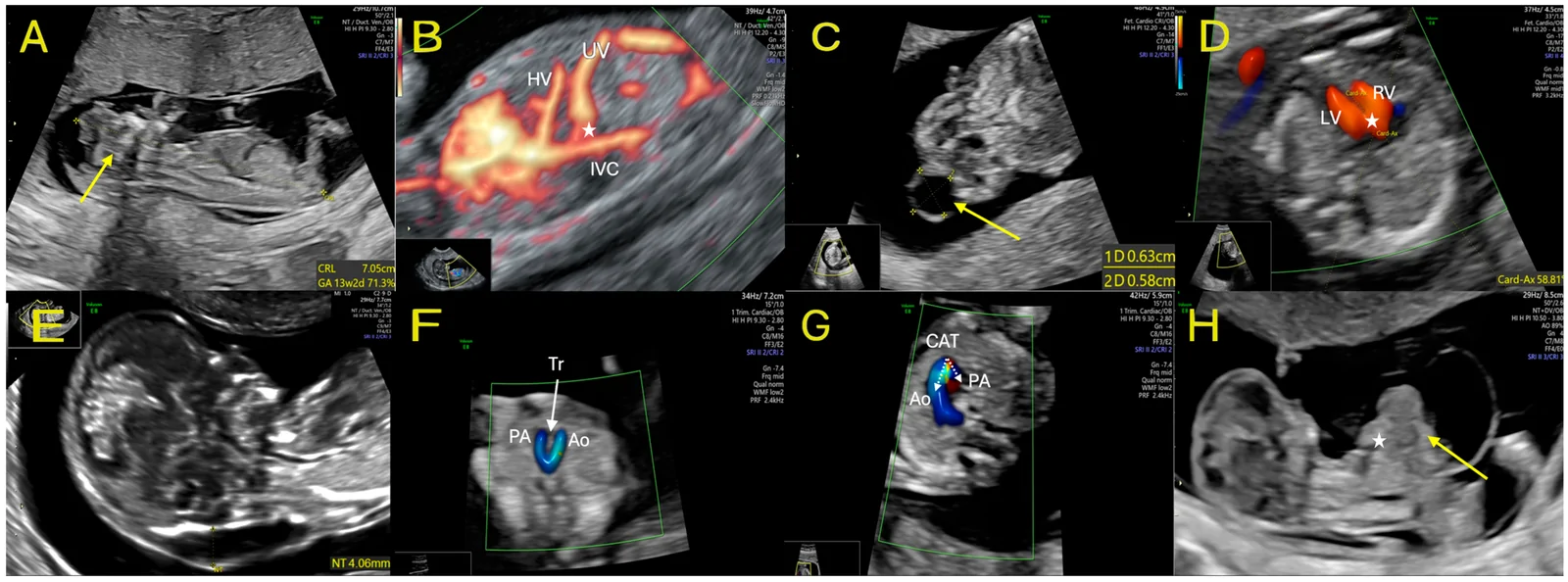
Spectrum of major structural anomalies and marker findings detected during the first-trimester ultrasound screening with anatomical protocol (11 + 0 to 13 + 6 weeks). (**A**) **Acrania/Exencephaly:** sagittal view of the entire fetus demonstrating the complete absence of the mineralized cranial vault above the orbits, with disorganized brain tissue directly exposed to the amniotic fluid (arrow) (**B**) **Agenesis of the ductus venosus:** color Doppler sagittal scan showing the complete absence of the ductus venosus with an anomalous intrahepatic thin connection (asterisk) draining directly into the inferior vena cava (**C**) **Sacrococcygeal teratoma:** axial view showing a large, well-defined mixed cystic-solid mass protruding from the fetal rump/sacral region (arrow) (**D**) **Atrioventricular septal defect (AVSD):** apical four-chamber view of the fetal heart with color Doppler mapping demonstrating complete atrioventricular septal defect. AVSD is demonstrated by the star (**E**) **Increased nuchal translucency:** sagittal scan of the fetus demonstrating an enlarged, fluid-filled space on the dorsal aspect of the fetal neck, measuring 4.06mm (>99th percentile) (**F**) **Right aortic arch (RAA):** axial three-vessel and trachea (3VT) view showing the aortic arch positioned to the right of the trachea, forming an abnormal "U-shaped" vascular configuration with the left-sided ductal arch (**G**) **Abnormal outflows/Common arterial trunk:** cardiac scan showing bifurcation of the common arterial trunk into the aorta and the pulmonary artery (**H**) **Pentalogy of Cantrell-** sagittal view of the fetal chest and abdomen with the high omphalocele (arrow) and ectopia cordis (asterisk) as a part of Pentalogy of Cantrell. **Abbreviations:** HV, hepatic vein; UV, umbilical vein; IVC, inferior vena cava; LV, left ventricle; RV, right ventricle; PA, pulmonary artery; Tr, trachea; Ao, aorta; CAT, common arterial trunk.

Cases were classified according to the primary affected organ system. Anomalies involving two or more organ systems were categorized as multiorgan anomalies. Among the 17 suspected cases, congenital cardiovascular anomalies constituted the largest diagnostic group, accounting for eight cases (47.1% of all suspected anomalies; 1.6% of the entire cohort). Central nervous system (CNS) anomalies were suspected in four cases (23.5% of all suspected anomalies; 0.8% of the total cohort), with an observational cluster of acrania/exencephaly constituting the majority of cases (*n* = 3). Multiple malformations were suspected in four fetuses (23.5% of suspected anomalies; 0.8% of the entire cohort). Additionally, one case of sacrococcygeal teratoma was diagnosed (0.2% of total cohort) (Figure 2).

### 3.3. Confirmation Rate and Follow-Up Outcomes

Of the 17 screen-positive cases, 14 were definitively confirmed on subsequent evaluations, yielding a confirmation rate of 82.4% (14/17; 95% CI: 56.6–96.2%) under a conservative analytical approach where the single case lacking diagnostic confirmation follow-up was retained in the denominator. Two cases proved to be false-positive findings (11.8%; 95% CI: 1.5–36.4%), and one patient was lost to follow-up prior to diagnostic confirmation (5.9%; 95% CI: 0.1–28.7%). To evaluate the robustness of this parameter, sensitivity analyses under alternative assumptions were performed:

- Excluding the single case lost to follow-up from the denominator yielded a confirmation rate of 87.5% (14/16; 95% CI: 61.7–98.4%).
- Explicitly classifying the lost-to-follow-up case as a false positive maintained the confirmation rate at 82.4% (14/17; 95% CI: 56.6–96.2%).

A comprehensive breakdown of the specific diagnostic categories, structural evolutions, and final outcomes demonstrated the following:

**Congenital Cardiac and Vascular Defects:** Cardiac anomalies constituted the largest group. Confirmed cases included an isolated right aortic arch with favorable postnatal outcome (Case 10), an unbalanced atrioventricular septal defect with right aortic arch and reversed ductus venosus flow resulting in intrauterine demise (Case 9). One case presenting with a suspected ventricular septal defect (VSD) or hypoplastic left heart syndrome (HLHS) associated with an increased nuchal translucency (NT) and abnormal karyotype (Case 17) was confirmed as a complex cardiac defect. Intrahepatic agenesis of the ductus venosus was prenatally suspected in two cases; this presented as a classic intrahepatic variant in Case 4, and an intrahepatic variant with a thin connection to the inferior vena cava in Case 5. Both cases were confirmed prenatally and resulted in live births. In one of these children (Case 5), an atrial septal defect (ASD) was diagnosed postnatally at one year of age and was successfully corrected surgically.

**Central Nervous System and Posterior Fossa:** An observational cluster of three acrania/exencephaly cases was identified (3/500 pregnancies, 0.6%). All three cases were definitively confirmed during early second-trimester assessments, subsequently leading to termination of pregnancy (TOP). Additionally, one case (Case 13) initially presented with a posterior fossa cyst and an abnormal intracranial translucency (IT) view; however, subsequent mid-trimester scans showed no phenotypic progression, resulting in the live birth of a healthy neonate.

**Multiorgan anomalies:** Four fetuses presented with multiorgan anomalies. These included a common arterial trunk with later detection of dysplastic pulmonary valve and agenesis of the corpus callosum (Case 11, outcome unknown); a case with early tricuspid regurgitation and reversed aortic flow that evolved into a complex phenotype with ventricular septal defect, ventriculomegaly and agenesis of the corpus callosum (Case 12, live birth); a fetus with interrupted retronasal triangle, megacystis and increased nuchal translucency (Case 14, lost to follow-up); and one case of Pentalogy of Cantrell confirmed on subsequent scans, resulting in intrauterine demise at 16–17 weeks (Case 16).

**Sacrococcygeal Teratoma (SCT):** One case of sacrococcygeal teratoma (Case 6) was detected during routine screening and subsequently confirmed on serial scans. Despite an attempted trial of in utero fetal therapy, the pregnancy ultimately resulted in intrauterine fetal demise (IUFD).

**False-Positives and Attrition:** Two cases with initially abnormal three-vessel-trachea view (Cases 7 and 8) resolved completely on second-trimester scans and resulted in healthy infants. One patient with suspected tetralogy of Fallot (Case 15) was lost to follow-up.

### 3.4. Nuchal Translucency and Statistical Associations

An increased nuchal translucency (NT) above the 95th percentile for gestational age was observed in 12 out of the 500 screened pregnancies (2.4%; 95% CI: 1.3–4.2%). This sonographic marker was more frequent among fetuses with confirmed major structural anomalies (3/14; 21.4%; 95% CI: 7.6–47.6%) than among the remaining pregnancies in the cohort (9/486; 1.85%; 95% CI: 1.0–3.5%) (*p* = 0.004, Fisher’s exact test). It should be noted that systematic outcome verification was not available for the screen-negative population. Therefore, the group of 486 pregnancies cannot be regarded as definitively free of major anomalies, and the comparison should be interpreted with appropriate caution.

## 4. Discussion

This study evaluated the diagnostic value of a systematic first-trimester fetal anatomical assessment using a standardized anatomical protocol in a single regional center. In a cohort of 500 consecutive singleton pregnancies from an unselected screening population, major structural anomalies were suspected in 3.4% of fetuses, with a baseline confirmation rate of 82.4% (14/17; 95% CI: 56.6–96.2%). These findings support the feasibility of performing an extended first-trimester anatomical examination outside tertiary referral centers. The overall proportion of suspected major structural anomalies (3.4%) is consistent with previous large-scale first-trimester screening studies in populations, where the prevalence of detected major structural anomalies typically ranged from 2.5% to 3.5% [3,6]. These results are further supported by nationwide screening data [23] and the most recent systematic review [7]. Importantly, our data reinforces the findings of Kenkhuis et al., confirming that the 11–14-week ultrasound scan cannot be replaced by cell-free DNA screening due to its critical, independent diagnostic value for detecting structural defects.

The overall confirmation rate of 82.4% aligns well with published data, where verification rates for anomalies suspected in the first trimester typically range from 75% to 90% [5,13,24]. Diagnostic discrepancies, including false-positive findings and one case lost to follow-up, reflect the inherent limitations of early fetal imaging, such as the small size of structures during organogenesis, spontaneous resolution of transient findings, and the evolutionary nature of certain anomalies. These observations underline the complementary roles of first- and second-trimester examinations.

A notable observation in our cohort was the identification of three cases of acrania/exencephaly (0.6%, or 1:167). Although this proportion appears higher than the background prevalence reported by EUROCAT and other European registries [25,26,27], the difference should be interpreted with caution. It most likely reflects a combination of methodological factors (first-trimester cohorts include early pregnancy losses and terminations that are often missing from birth registries) and random clustering within a modest sample size. We therefore present these cases primarily as an observational cluster rather than evidence of increased population prevalence. All three cases were confirmed in the early second trimester, illustrating that severe neural tube defects can be reliably recognized between 11 + 0 and 13 + 6 weeks when systematic anatomical landmarks are assessed [28,29,30,31,32].

Early neurosonography also revealed its limitations. In one case (Case 13), an abnormal intracranial translucency and suspected posterior fossa cyst resolved completely on mid-trimester follow-up, resulting in a healthy neonate. This underscores that first-trimester posterior fossa findings require careful serial evaluation before definitive conclusions are drawn [33,34,35].

Congenital heart and vascular defects constituted the largest diagnostic group in our cohort (8 cases, 47.1% of suspected anomalies), reflecting global epidemiological patterns where congenital heart defects (CHDs) represent the most frequent major structural malformations [6,36,37]. Our findings align with work by Popa et al. [36] and Duta et al. [38], demonstrating the efficacy of systematic first-trimester cardiac screening in unselected cohorts. In line with national data from Poland [39,40,41], early functional alterations and Doppler wave abnormalities served as critical red flags. This was clearly demonstrated in Case 12, where initial tricuspid regurgitation and reversed aortic arch flow preceded the full mid-trimester structural manifestation of a complex multiorgan phenotype (VSD, ventriculomegaly, agenesis of the corpus callosum). This sequential clinical manifestation underscores an important diagnostic principle: early functional cardiac anomalies or altered Doppler waveform patterns may precede the full anatomical expression of both cardiac and extracardiac defects.

Conversely, our extended protocol facilitated the detection of both benign anatomical variants (isolated right aortic arch, Case 10) and complex venous malformations (absent ductus venosus with direct thin intrahepatic drainage into the inferior vena cava, Case 5). In Case 5, an associated atrial septal defect (ASD) was diagnosed postnatally at one year of age and successfully repaired. Because the foramen ovale remains physiologically patent in utero, isolated ASDs cannot be detected prenatally. This case illustrates both the evolutionary nature of certain cardiac lesions and the necessity of incorporating formal postnatal follow-up into first-trimester diagnostic protocols [42,43,44].

Rare and severe malformations, such as sacrococcygeal teratoma (Case 6) and Pentalogy of Cantrell (Case 16), further validate the utility of evaluating the entire fetal contour, abdominal wall, and lower pole [45,46,47,48,49,50,51,52]. Early detection of dynamic lesions like sacrococcygeal teratoma is of high clinical value, allowing timely parental counseling, early fetal echocardiography to monitor for high-output cardiac failure, and prompt referral to tertiary centers for multidisciplinary perinatal management. Similarly, multi-system red flags—such as the interrupted retronasal triangle view and early megacystis observed in Case 14—demonstrate the necessity of moving beyond isolated nuchal translucency (NT) measurement toward an integrated, systematic multi-system anatomical survey [53].

### 4.1. The Value of a First-Trimester Anatomical Protocol

The implementation of a systematic anatomical protocol is one of the main strengths of this study. This approach is strongly supported by contemporary literature, which highlights the added diagnostic value of detailed first-trimester ultrasound, even in the era of widespread cell-free DNA (cfDNA) screening [3,14]. Importantly, even in pregnancies with low-risk or normal cfDNA results, structured early ultrasound evaluation remains essential, as clinically significant non-chromosomal anomalies are frequently identified [23,54,55].

A structured first-trimester protocol improves the early detection of major structural anomalies, particularly those affecting the cardiovascular and central nervous systems. The incorporation of early neurosonography enables reliable diagnosis of severe CNS defects already at 11–13 + 6 weeks [28,35]. Likewise, standardized fetal echocardiography protocols [24] combined with assessment of nuchal translucency, ductus venosus flow, and tricuspid regurgitation [56] can enhance the efficacy of cardiac evaluation.

With regard to cardiac anomalies, a comprehensive meta-analysis by Karim et al. demonstrated that the combination of the four-chamber view, outflow tract views, and color Doppler mapping achieves the highest sensitivity for detecting major heart defects in the first trimester, reaching 80.0% (95% CI: 67.9–89.8%) [57]. This evidence strongly supports the methodological framework applied in our study. Our results are consistent with large-scale population-based data showing that detailed first-trimester anatomical protocols can increase early detection rates of major structural anomalies [11]. Crucially, a substantial proportion of these anomalies occur in fetuses with normal nuchal translucency [6,58], underscoring the limitations of relying solely on NT measurement. These findings align with both pioneering screening frameworks proposed by Nicolaides [59] and current ISUOG practice guidelines, which increasingly endorse detailed anatomical assessment, including cardiac evaluation, between 11 + 0 and 13 + 6 weeks of gestation [60].

In Poland, the second-trimester scan remains the formal standard for structural anomaly screening according to PTGiP recommendations [18,20]. Our study, reporting a 3.4% prevalence of suspected structural anomalies in an unselected regional population, suggests that systematic anatomical screening is feasible beyond leading academic centers when performed under favorable conditions. This supports the broader international trend toward earlier and more comprehensive prenatal diagnosis [61].

### 4.2. Strengths and Limitations

The principal strength of this study is its conduct in a regional center serving an unselected population, in contrast to most previous reports from highly selected cohorts in referral centers. The use of a structured first-trimester anatomical protocol, high-end ultrasound equipment, adequate examination time, and performance of all scans by a single experienced operator ensured both high methodological consistency and high confirmation rate among screen-positive cases.

Several limitations must be acknowledged. First, the retrospective, single-center design and the fact that all examinations were performed by a single experienced operator limit the generalizability of the findings to routine practice with operators of varying experience and preclude assessment of inter-observer variability. Second, systematic follow-up was not available for screen-negative pregnancies, preventing the ascertainment of potential false-negative cases; consequently, formal diagnostic performance parameters (such as sensitivity and specificity) were deliberately not derived. Regarding missing follow-up among screen-positive fetuses, one case was lost prior to verification, though our sensitivity analysis demonstrated robust confirmation rates across assumptions (82.4% vs. 87.5%). Third, the sample size (*n* = 500) limits precise estimates for extremely rare anomalies, and although genetic testing was routinely recommended in eligible cases, not all patients opted to undergo invasive or non-invasive testing. Finally, due to ongoing organogenesis, certain subtle anomalies remain inherently undetectable in the first trimester.

An important consideration when implementing detailed first-trimester screening is safety of Doppler techniques before 14 weeks of gestation [14,62]. Color Doppler mapping is generally considered safe between 11 + 0 and 13 + 6 weeks, provided that the ALARA (*as low as reasonably achievable*) principle is strictly respected [63,64,65]. Beyond safety, major barriers to widespread adoption include the need for advanced operator training, sufficient examination time, and access to high-resolution equipment. In practice, most studies use a combined approach, beginning with transabdominal sonography (TAS) and supplementing with transvaginal sonography (TVS) when visualization is suboptimal [66]. We believe the choice of ultrasound modality should remain flexible, tailored to clinician expertise, patient preference, and individual body habitus, particularly in cases of maternal obesity [67].

### 4.3. Clinical Implications and Future Research Directions

Congenital anomalies affect approximately 1 in 33 neonates globally and cause roughly 240,000 neonatal deaths annually [2,68]. In this context, early detection of structural anomalies using extended first-trimester ultrasound protocols assumes profound significance. Our findings support the potential clinical value of expanding detailed first-trimester anatomical assessment in regional settings. This is particularly relevant in regional centers with limited access to dedicated, on-site maternal-fetal medicine specialists. The early detection of major fetal anomalies necessitates sensitive, individualized counseling. Receiving news of a fetal anomaly generates severe emotional distress for parents; however, timely, honest, and supportive communication from the medical team plays a critical role in the adaptation and crisis-management process [69,70,71]. This is particularly vital for vulnerable subgroups, including pregnant individuals with pre-existing mental health conditions, who face unique challenges when navigating unexpected prenatal diagnoses [72]. Furthermore, long-term studies indicate that the manner in which a prenatal diagnosis is delivered significantly influences the severity of parental traumatic stress, long-term psychological resilience, and relationship satisfaction in subsequent years [73,74]. Future research should involve larger, prospective multicenter cohorts across different regions of Poland, with systematic long-term follow-up of both screen-positive and screen-negative pregnancies. Particular attention should be paid to the standardization of extended first-trimester training protocols, assessment of inter-observer reproducibility, and evaluation of diagnostic performance (including sensitivity) in routine clinical practice involving operators of varying experience. Studies addressing cost-effectiveness, resource requirements, and the psychological impact of early anomaly detection on patients and their partners will also be essential. Such data are needed before any broader implementation of detailed first-trimester anatomical assessment into the national perinatal care system can be considered.

## 5. Conclusions

This study demonstrates that the integration of a systematic first-trimester anatomical protocol into routine screening can provide diagnostic value even in a regional center serving an unselected population. A comprehensive multi-system evaluation performed between 11 + 0 and 13 + 6 weeks enabled the early detection of a clinically relevant proportion of major structural anomalies, particularly lethal central nervous system defects and complex congenital heart defects. Although first-trimester anatomical screening does not replace the established mid-trimester scan, it can meaningfully advance the timeline of prenatal diagnosis when performed under favorable conditions. Early identification facilitates informed parental counseling, optimized pregnancy management, and timely referral to tertiary centers when indicated. Our real-world data support the feasibility of structured first-trimester protocols in a regional setting, provided that examinations are performed by adequately trained operators using high-resolution equipment. In the Polish context, these findings align with the ongoing evolution of prenatal care and may encourage a carefully phased exploration of more detailed first-trimester assessment, while maintaining the second-trimester scan as the current standard according to PTGiP guidelines. Future multicenter studies with systematic follow-up are needed to evaluate the true diagnostic performance (including sensitivity), cost-effectiveness, training requirements, inter-observer reproducibility, and long-term outcomes of this approach. In conclusion, systematic first-trimester examination of fetal anatomy appears to be a useful complementary tool for early risk stratification, but its performance and generalizability require further prospective validation.

## Figures and Tables

**Table 1:** Anatomical structures and parameters evaluated in first-trimester screening in the anatomical protocol.

Category	Parameters Evaluated
Biometry & Dating	CRL, pregnancy dating
Nuchal region	NT measurement
Face	Nasal bone, facial profile, orbits, lips, retronasal triangle
Heart	4CV, OTV, CF Doppler, cardiac axis
Venous flow	DV PI
Doppler	UtA Doppler
Maternal factors	Mean arterial pressure, cervical length
Biochemistry & Risk	Free β-hCG, PAPP-A, PlGF; combined risk for trisomy 21/18/13, PE, FGR
Central nervous system	Cranium shape, brain structures (butterfly sign), third ventricle, cerebral aqueduct (of Sylvius), IT, posterior fossa
Spine	Vertebrae (longitudinal and axial), intact overlying skin
Abdominal wall	Abdominal wall integrity, umbilical cord insertion
Abdomen	Stomach, kidneys, bladder
Limbs	Presence of all 4 limbs, hands, feet
Placenta & Cord	Size and texture, three-vessel cord

**CRL**, crown-rump length; **NT**, nuchal translucency; **4CV**, four-chamber view; **CF**, color flow; **OTV**, outflow-tract view; **DV**, ductus venosus; **PI**, pulsatility index; **UtA,** uterine artery; **β-hCG**, beta-human chorionic gonadotropin; **PAPP-A**, pregnancy-associated plasma protein A; **PlGF**, placental growth factor; **PE**, preeclampsia; **FGR**, fetal growth restriction; **IT**, intracranial translucency.

**Table 2:** Maternal characteristics of the study population (*n* = 500).

Variable	*n* (%) or Mean ± SD
**Age (years)**	30.7 ± 5.1
**BMI (kg/m^2^)**	24.8 ± 4.7
**Parity**	
Nulliparous	156 (31.2%)
Multiparous	344 (68.8%)
**Mode of conception**	
Spontaneous	498 (99.6%)
ART (Assisted Reproductive Technology)	2 (0.4%)
**Chronic medical conditions**	
Hypertension	9 (1.8%)
Diabetes mellitus	7 (1.4%)
**Smoking status**	
*Non-smoker*	479 (95.8%)
*Smoking history*	21 (4.2%)
- Current smoker	6 (28.6%)
- Former smoker	15 (71.4%)

**Table 3:** Medical details of major fetal structural anomalies suspected on first- trimester ultrasound screening with anatomical protocol (*n* = 17).

Case	Medical History	NT (mm)	NB	TR	DV (PVIV)	4CH	3VTV	Fetal Anatomy	Risk of Aneuploidy	Post-Test Referral	Prenatal Genetic Testing & Results	Verification of Diagnosis	Fetal Outcome
1	Age 36 GI Low risk group BMI 23	1.9	Hypoplastic	+	0.83	Normal	Normal	Acrania/exencephaly	T21 1:203 T18 1:449 T13 1:1271	No	No	Yes	ToP
2	Age 33 GII PII Low risk group BMI 22	1.00	Hypoplastic	+	0.74	Normal	Normal	Acrania/exencephaly	T21 1:8394 T18 < 1:20,000 T13 1:3231	No	No	Yes	ToP
3	Age 29 GI Low-risk group BMI 34	1.1	Hypoplastic	+	1.09	Incorrect cardiac axis	Normal	Acrania/exencephaly	T21 1:3401 T18 1:4920 T13 1:8751	No	No	Yes	ToP
4	Age 21 GI Maternal smoking BMI 30	1.71	+	+	Agenesis of DV (intrahepatic type)	Normal	Normal	No other anomalies detected	T21 < 1: 20,000 T18 < 1:20,000 T13 < 1:20,000	Fetal echocardiography	No	Yes	Healthy live birth
5	Age 34 GII PII Low risk group BMI 21	1.33	+	+	Agenesis of DV (intrahepatic with thin connection to IVC)	Normal	Normal	No other anomalies detected	T21 1:724 T18 1:2377 T13 1:1780	Fetal echocardiography	No	Yes	Live birth (with postnatal ASD)
6	Age 26 GII PII Low risk group BMI 34	1.6	+	+	1.04	Normal	Normal	Sacrococcygeal teratoma	T21 1:17,593 T18 < 1:20,000 T13 < 1:20,000	Genetic counseling	Normal karyotype	Yes	Attempted intrauterine therapy, fetal demise
7	Age 34 GII PII Low risk group BMI 23	2.5	+	+	1.07	Normal	Abnormal	No other anomalies detected	T21 1:1807 T18 1:11,850 T13 < 1:20,000	Fetal echocardiography	No	False positive	Healthy live birth
8	Age 29 GII PII Low risk group BMI 33	4.8	+	+	1.01	Incorrect cardiac axis	Abnormal	No other anomalies detected	T21 > 1:4 T18 1:277 T13 1:1916	Genetic counseling	Normal karyotype	False positive	Healthy live birth
9	Age 34 GIV PIII Low risk group BMI 35	6.9	Hypoplastic	+	1.81 reversed flow	AVSD	RAA	No other anomalies detected	T21 > 1:4 T18 1:467 T13 1:168	Fetal echocardiography, genetic counseling	Recommended, not performed	Yes	Intrauterine demise
10	Age 26 GI Low risk group BMI 23	1.74	+	+	0.95	Normal	RAA	No other anomalies detected	T21 1:18,670 T18 < 1:20,000 T13 < 1:20,000	Fetal echocardiography	Recommended	Yes	Live birth (isolated anomaly)
11	Age 29 GIII PII Low risk group BMI 22	1.8	+	Not evaluable	0.98	Incorrect cardiac axis	CAT	No other anomalies detected	T21 1:3007 T18 1:5912 T13 < 1:20,000	Fetal echocardiography	Recommended	Yes (additional dysplastic PV and ACC)	Pregnancy outcome unknown
12	Age 29 GIV PIII Maternal smoking BMI 20	2.3	+	Regurgitation	0.82	Normal	Normal	Reversed flow in aorta (3VV)	T21 1:414 T18 1:1841 T13 1:34	Fetal echocardiography, genetic counseling	Normal karyotype	Yes (additional VSD, VM, ACC	Live birth
13	Age 34 GIII PIII Low risk group BMI 35	1.8	+	+	1.24	Normal	Normal	PFC, abnormal IT	T21 1:1497 T18 1:15,684 T13 < 1:20,000	No	No	Yes transitory	Healthy live birth
14	Age 32 GIII PII Low risk group BMI 21	3.4	+	+	1.42	Normal	Normal	Interrupted RT, megacystis, DUT	T21 1:21 T18 1:10 T13 1:10	Genetic counseling	Recommended	Yes	Pregnancy outcome unknown
15	Age 28 GII PII Low risk group BMI 20	1.8	+	+	1.06	Incorrect cardiac axis	Abnormal outflows (suspicion of TOF)	No other anomalies detected	T21 1:1346 T18 < 1:20,000 T13 1:8199	Fetal echocardiography	No	Lost to follow-up	Lost to follow-up
16	Age 39 GII PII Low risk group BMI 21	1.8	Hypoplastic	Not evaluable	Not evaluable	Ectopia cordis	Ectopia cordis	Omphalocele, LSD, DPD, ADD-(POC), PFC, interrupted RT	T21 1:363 T18 >1:4 T13 1:10	Genetic counseling	Recommended	Yes	Intrauterine demise
17	Age 29 GII PII Low risk group BMI 33	4.1	Hypoplastic	+	Reversed flow	RV > LV single ventricular inflow	Abnormal	Maxillary gap	T21 >1:4 T18 1:12 T13 1:101	Fetal echocardiography, genetic counseling	46,XX,der(3)t(1;3) (trisomy 1q32 and monosomy 3q29)”	Yes (complex cardiac defect) VSD, HLHS	Termination of pregnancy

**Abbreviations: ToP**, termination of pregnancy; **3VTV**, three-vessel and trachea view; **3VV**, three-vessel view; **4CH**, four-chamber view; **ACC,** agenesis of corpus callosum; **ADD,** anterior diaphragmatic defect; **AVSD**, atrioventricular septal defect; **BMI**, body mass index; **CAT**, common arterial trunk; **DPD,** diaphragmatic pericardial defect; **DUT**, dilated urinary tracts; **DV (PVIV),** ductus venosus (pulsatility index for veins); **G**, gestation; **HLHS**, hypoplastic left heart syndrome; **IT**, intracranial translucency; **IVC,** inferior vena cava; **LSD,** lower sternal defect; **LV**, left ventricle; **NB,** nasal bone; **NT**, nuchal translucency; **P**, pregnancy; **PFC**, posterior fossa cyst; **POC,** Pentalogy of Cantrell; **PV,** pulmonary valve; **RAA**, right aortic arch; **RT**, retronasal triangle; **RV**, right ventricle; **TOF**, Tetralogy of Fallot; **TR,** tricuspid regurgitation; **VM**, ventriculomegaly; **VSD**, ventricular septal defect.

## Data Availability

The raw data supporting the conclusions of this article will be made available by the authors on request. The data are not publicly available due to privacy and ethical restrictions regarding patient medical records.
